# Supplementary material for: Field testing two existing, standardized respiratory severity scores (LIBSS and ReSViNET) in infants presenting with acute respiratory illness to tertiary hospitals in Rwanda – a validation and inter-rater reliability study
Source: PLoS One. 2021 Nov 4;16(11):e0258882. doi: 10.1371/journal.pone.0258882 (PMC8568200; doi:10.1371/journal.pone.0258882)
Supplement: S1 File — (DOCX) [file pone.0258882.s001.docx]

**Location in manuscript**

Item 1: See Title

Item 2: See abstract

Item 3a: See Introduction paragraph 4

Item 3b: See Introduction “Objectives”

Item 4a: See methods – “Study sites (data source)”

Item 4b: “study design”

Item 5a: See methods – “Study sites (data source)”

Item 5b: See methods – “Study population”

Item 5c: See methods – “Clinical care of infants”

Item 6a: See methods – “Outcomes, data management and statistical methods”

Item 6b: See methods – “Clinical care of infants”

Item 7a/b: See methods – “Data collection tool”

Item 8: See methods – “Sample size”

Item 9: See results – “Data quality”

Item 10a/b/c/d: See methods – “Outcomes, data management and statistical methods”

Item 10e – not applicable

Item 11: not applicable

Item 12: not applicable

**Location in manuscript**

Item 13a: See Results – “Figure 1”

Item 13b: See Results – “Table 2”

Item 13c: Not applicable

Item 14a/b: Not applicable

Item 15a/b: Not applicable

Item 16: See Results – Table 5-6 and Figures 2-3

Item 17: Not applicable

Item 18: See Discussion – “Limitations”

Item 19a: See Discussion – “Validity of the scores”

Items 19b and 20: See Discussion – “Conclusion”

Item 21: not available

Item 22: See ETHICAL CONSIDERATIONS AND DECLARATIONS “Funding & Sponsors”
