# Supplementary material for: Field testing two existing, standardized respiratory severity scores (LIBSS and ReSViNET) in infants presenting with acute respiratory illness to tertiary hospitals in Rwanda – a validation and inter-rater reliability study
Source: PLoS One. 2021 Nov 4;16(11):e0258882. doi: 10.1371/journal.pone.0258882 (PMC8568200; doi:10.1371/journal.pone.0258882)
Supplement: S2 File — (DOCX) [file pone.0258882.s002.docx]

| **Liverpool Infant Bronchiolitis Severity Score: Infant aged under three months**  **/Abana bafite munsi y’ amezi atatu** | | | | | | | | | |
| --- | --- | --- | --- | --- | --- | --- | --- | --- | --- |
| Day of illness:  Iminsi y’ubarwayi | | | | | | | | | |
| 1. Do you have any concerns relating to the infant’s overall condition?   Hari ikibazo ufite kijyanye nuko mwana ameze? | | | | | | | | | |
| No concerns ntakibazo  (condition is stable or improving/ ameze neza cg arikoroherwa) | | 0 | | |  | | | Comments:  ibisobanuro | |
| Some concerns Dufite impungenge (may become unstable/requires close observation/ ashobora kuremba/akeneye gukurikiranirwa hafi ) | | 4 | | |  | | |  |  |
| Extremely concerned (unstable requires immediate medical review)  Turahangayitse cyane (Ararembye akeneye guhita avurwa) | | 8 | | |  | | |  |  |
| 2. Apnoea guhagarika guhumeka | | | | | | | | | |
| None  Ntanarimwe | | 0 | | |  | | | Comments:  ibisobanuro | |
| Occasional self-correcting apnoea / short pauses  Ahagarara guhumeka bikosora /guhagarika guhumeka akanya gato | | 2 | | |  | | |  |  |
| Apnoea’s increasing frequency & duration  guhagarika guhumeka byiyongera mu nshuro n’igihe bimara | | 4 | | |  | | |  |  |
| Apnoea’s requiring stimulation  Guhagarara guhumeka biri gusaba kumukoraho ngo yongere ahumeke | | 6 | | |  | | |  |  |
| Apnoea’s requiring bag & mask ventilation  Guhagara guhumeka bikeneye kumubaginga ngo yongere ahumeke | | 8 | | |  | | |  |  |
| 3. Increased work of breathing/ kongera imbaraga ahumekesha (Absent or Mild =0) Please complete all boxes / uzuza udusanduku twose kuri iki gice | | | | | | | | | |
| Moderate/severe recession  Biringaniye/ bikabije mugukoresha imbaraga zo guhumeka | 0 | | | 2 | | |  | | Comments:  Ibisobanuro |
| Moderate/severe tracheal tug  iringaniye/ ikabije tracheal tug | 0 | | | 2 | | |  | |  |
| Moderate/severe nasal flare  Biringaniye/ bikabije muguhumekesha amazuru | 0 | | | 2 | | |  | |  |
| Moderate/severe head bobbing  Kuzunguza umutwe | 0 | | | 4 | | |  | |  |
| Grunting  Kuniha | 0 | | | 4 | | |  | |  |
| Central cyanosis (blue lips / tongue)  Guhinduka ubururu ( iminwa/ ururimi bisa n’ubururu) | 0 | | | 6 | | |  | |  |
| 4. % oxygen to maintain saturations >92% (or usual saturation level if infant has congenital heart defect)  Umwuka wa ogisijene ukenewe ngo saturation igume heuru ya 92%( cg saturasiyo fatizo mubana bafite uburwayi bw’umutima | | | | | | | | | |
| 21% (room air umwuka usanwe dumeka) | | 0 | | |  | | | Comments:  Ibisobanuro | |
| 22 - 40% (0.02 - 6L/min) | | 2 | | |  | | |  |  |
| 41 - 50% (7 - 10L/min) | | 4 | | |  | | |  |  |
| >50% (>10L/min) | | 6 | | |  | | |  |  |
| Actual amount of oxygen administered  Igipimo cya oxygen yatanzwe | | | | |  | | |  |  |
| Mode of oxygen delivery/uburyo uxygen itangwa: Nasal specs (NS); Face Mask (FM); Head box (HB); HiFlow (HF); nCPAP (CP) | | | | |  | | |  |  |
| 5. Respiratory rate (breaths per minute)  Inshuro ahumeka mu munota | | | | | | | | | |
| 25 - 59 | | 0 | | |  | Comments:  Ibisobanuro | | | |
| 60 - 70 | | 2 | | |  |  |  |  |  |
| <25 or >70 | | 4 | | |  |  |  |  |  |
| 6. Heart rate (beats per minute)  Inshuro umutima utera mu munota | | | | | | | | | |
| 105 - 165 | | 0 |  | | | Comments:  Ibisobanuro | | | |
| 166 - 180 | | 2 |  | | |  |  |  |  |
| <105 or >180 | | 4 |  | | |  |  |  |  |
| 7. Appearance  Uko agaragara | | | | | | | | | |
| Alert & active / normal sleep  Arakangutse & afite imbaraga / arasinziriye bisanzwe | | 0 | | |  | | | Comments:  Ibisobanuro | |
| Irritable / fractious / restless  Afite amahane/ arikurira cyane/ intege nke | | 2 | | |  | | |  |  |
| Floppy / lethargic / poor interaction  Yacitse intege cyane/ararembye | | 4 | | |  | | |  |  |
| Only responds to pain / unresponsive  Asubiza kububabare bwonyine/ cyangwa ntasubiza | | 6 | | |  | | |  |  |
| AVPU SCORE | | | | |  | | |  |  |
| 8. Feeding  Kugaburira | | | | | | | | | |
| >75% or normal amount of feeds via usual route  Afata ibyokurya byuzuye cyangwa hejuru ya 75% byiko akwiye gufata. | | 0 | | |  | | | Comments:  Ibisobanuro | |
| 50 - 75% of feeds of normal feeds via usual route  Arya 50-75% by’ibyo kurya akwiye gufata | | 2 | | |  | | |  |  |
| <50% of feeds or needing NG feeds / IV fluids  Abona munsi ya 50% byibyokurya bisanzwe cyangwa akeneye kugaburirwa binyuze muri sonde cyangwa mu mutsi | | 4 | | |  | | |  |  |
| 9. Urine output | | | | | | | | | |
| Usual number of wet nappies (> 2 mLs /kg/hr)  Umubare usanzwe wa Diapers | | 0 | | |  | | | Comments:  Ibisobanuro | |
| Reduction in number of wet nappies (1 - 2 mLs /kg/hr)  Umubare wa Diapers wagabanutse | | 2 | | |  | | |  |  |
| Small volumes of concentrated urine / no urine (< 1mL/kg/hr)  Afite inkari nke ziri concentre/ nta nkari afite | | 4 | | |  | | |  |  |
| 10. Central capillary refill time (preferably press on the sternum for 5 seconds) | | | | | | | | | |
| < 2 seconds | | 0 | | |  | | | Comments:  Ibisobanuro | |
| > 2 seconds | | 2 | | |  | | |  |  |
| Actual capillary refill time in seconds | | | | |  | | |  |  |

| LIBSS Score Total:  Mild (0-10); Moderate (11-20); Severe (> 21)  Guhumeka nabi byoroshe (0-10), guhumeka nabi biringaniye (11-20), guhumeka nabi bikabije (>21) |  | Comments:  Ibisobanuro |
| --- | --- | --- |
