# Supplementary material for: Field testing two existing, standardized respiratory severity scores (LIBSS and ReSViNET) in infants presenting with acute respiratory illness to tertiary hospitals in Rwanda – a validation and inter-rater reliability study
Source: PLoS One. 2021 Nov 4;16(11):e0258882. doi: 10.1371/journal.pone.0258882 (PMC8568200; doi:10.1371/journal.pone.0258882)
Supplement: S3 File — (DOCX) [file pone.0258882.s003.docx]

| **Liverpool Infant Bronchiolitis Severity Score:**  **children aged three months and over/ Abana bafite amezi atatu gusubiza hejuru** | | | | | | | |
| --- | --- | --- | --- | --- | --- | --- | --- |
| **Day of illness:**  **Iminsi y’ubarwayi** | | | | | | | |
| 1. **Do you have any concerns relating to the infant’s overall condition?**   **Hari ikibazo ufite kijyanye nuko umwana ameze?** | | | | | | | |
| No concerns ntakibazo (condition is stable or improving/ ameze neza cg arikoroherwa) | | **0** | |  | | **Comments:**  **ibisobanuro** | |
| Some concerns (may become unstable/requires close observation)  Dufite impungenge ( ashobora kuremba/akeneye gukurikiranirwa hafi) | | **4** | |  | |  |  |
| Extremely concerned (unstable requires immediate medical review)  Turahangayitse cyane (Ararembye akeneye guhita avurwa) | | **8** | |  | |  |  |
| **2. Apnoea guhagarika guhumeka** | | | | | | | |
| None  Ntanarimwe | | **0** | |  | | **Comments:**  **ibisobanuro** | |
| Occasional self-correcting apnoea / short pauses  Ahagarara guhumeka byikosora /guhagarika guhumeka akanya gato | | **2** | |  | |  |  |
| Apnoea’s increasing frequency & duration  guhagarika guhumeka byiyongera mu nshuro n’igihe bimara | | **4** | |  | |  |  |
| Apnoea’s requiring stimulation  Guhagarara guhumeka biri gusaba kumukoraho ngo yongere ahumeke | | **6** | |  | |  |  |
| Apnoea’s requiring bag & mask ventilation  Guhagara guhumeka bikeneye kumuhumekesha hakoreshejwe Ambubag. | | **8** | |  | |  |  |
| **3. Increased work of breathing (Absent or mild =0) Please complete all boxes in this section**  **kongera imbaraga ahumekesha (ntazo cg nkeya =0) uzuza udusanduku twose kuri iki gice** | | | | | | | |
| Moderate/severe recession  Biringaniye/ bikabije mugukoresha imbaraga zo guhumeka | **0** | **2** | |  | | **Comments:**  **ibusobanuro** | |
| Moderate/severe tracheal tug  iringaniye/ ikabije tracheal tug | **0** | **2** | |  | |  |  |
| Moderate/severe nasal flare  Biringaniye/ bikabije muguhumekesha amazuru | **0** | **2** | |  | |  |  |
| Moderate/severe head bobbing  Kuzunguza umutwe | **0** | **4** | |  | |  |  |
| Grunting  Kuniha | **0** | **4** | |  | |  |  |
| Central cyanosis (blue lips / tongue)  Guhinduka ubururu ( iminwa/ ururimi bisa n’ubururu) | **0** | **6** | |  | |  |  |
| **4. % oxygen to maintain saturations >92% (or usual saturation level if infant has congenital heart defect)**  **Umwuka wa ogisijene ukenewe ngo saturation igume heuru ya 92%( cg saturasiyo fatizo mubana bafite uburwayi bw’umutima )** | | | | | | | |
| 21% (room air , umwuka usanwe dumeka) | | **0** | |  | | **Comments:**  **ibusobanuro** | |
| 22 - 40% (0.02 - 6L/min) | | **2** | |  | |  |  |
| 41 - 50% (7 - 10L/min) | | **4** | |  | |  |  |
| >50% (>10L/min) | | **6** | |  | |  |  |
| Actual amount of oxygen administered  Igipimo cya ogisijene yatanzwe | | | |  | |  |  |
| Mode of oxygen delivery/ uburyo ogisijene itangwa : Nasal specs (NS); Face Mask (FM); Head box (HB); HiFlow (HF); nCPAP (CP) | | | |  | |  |  |
| **5. Respiratory rate (breaths per minute)**  **Inshuro ahumeka mu munota** | | | | | | | |
| 20 – 55 | | **0** | |  | **Comments:**  **ibusobanuro** | | |
| 56 – 65 | | **2** | |  |  |  |  |
| <20 or >65 | | **4** | |  |  |  |  |
| **6. Heart rate (beats per minute)**  **Inshuro umutima utera mu munota** | | | | | | | |
| 95 – 145 | | **0** | |  | **Comments:**  **ibusobanuro** | | |
| 146 – 160 | | **2** | |  |  |  |  |
| <95 or >160 | | **4** | |  |  |  |  |
| **7. Appearance**  **Uko agaragara** | | | | | | | |
| Alert & active / normal sleep  Arakangutse & arakina / arasinziriye bisanzwe | | **0** | |  | | **Comments:**  **ibisobanuro** | |
| Irritable / fractious / restless  Afite amahane/ arikurira cyane / ntagobwo atuje | | **2** | |  | |  |  |
| Floppy / lethargic / poor interaction  Yacitse intege cyane/ararembye / ntabasha gukina | | **4** | |  | |  |  |
| Only responds to pain/unresponsive  Asubiza kububabare bwonyine/ ntasubiza | | **6** | |  | |  |  |
| AVPU Score | | | |  | |  |  |
| **8. Feeding**  **Kugaburira** | | | | | | | |
| >75% of feeds or normal amount of feeds via usual route  Afata ibyokurya byuzuye cyangwa hejuru ya 75% byibyo akwiye gufata. | | **0** | |  | | **Comments:**  **Ibisobanuro** | |
| 50 - 75% of feeds via usual route  Arya 50-75% by’ibyo kurya akwiye gufata | | **2** | |  | |  |  |
| <50% of feeds or needing NG feeds / IV fluids  Abona munsi ya 50% byibyokurya bisanzwe cyangwa akeneye kugaburirwa binyuze muri sonde cyangwa mu mutsi | | **4** | |  | |  |  |
| **9. Urine output**  **Inkali yihagarika** | | | | | | | |
| Usual number of wet nappies (> 2 mLs /kg/hr)  Umubare usanzwe wa pampegisi | | **0** | |  | | **Comments:**  **ibisobanuro** | |
| Reduction in number of wet nappies (1 - 2 mLs /kg/hr)  Umubare wa pampegisi wagabanutse | | **2** | |  | |  |  |
| Small volumes of concentrated urine / no urine (< 1mL/kg/hr)  Afite inkari nke ziri consantere/ nta nkari afite | | **4** | |  | |  |  |
| **10.Central capillary refill time (preferably press on the sternum for 5 seconds)** | | | | | | | |
| < 2 seconds | | 0 | |  | | **Comments:**  **Ibisobanuro** | |
| > 2 seconds | | 2 | |  | |  |  |
| Actual capillary refill time in seconds | | | |  | |  |  |
| **LIBSS Score Total**  Mild (0-10); Moderate (11-20); Severe (>21)  Guhumeka nabi byoroshe (0-10), guhumeka nabi biringaniye (11-20), guhumeka nabi bikabije (>21) | | |  | | **Comments:**  **Ibisobanuro** | |  |
