# Supplementary material for: Field testing two existing, standardized respiratory severity scores (LIBSS and ReSViNET) in infants presenting with acute respiratory illness to tertiary hospitals in Rwanda – a validation and inter-rater reliability study
Source: PLoS One. 2021 Nov 4;16(11):e0258882. doi: 10.1371/journal.pone.0258882 (PMC8568200; doi:10.1371/journal.pone.0258882)
Supplement: S4 File — (DOCX) [file pone.0258882.s004.docx]

**ReSVinet scale.**

|  | **Item** | **0 points** | **1 points** | **2 points** | **3 points** |
| --- | --- | --- | --- | --- | --- |
|  | **Uduce** | **0 amanota** | **inota rimwe** | **amanota 2** | **amanota 3** |
| 1 | Feeding intolerance | No | Mild Decreased appetite and/or isolated vomits with cough. | Partial Frequent vomits with cough, rejected feed but able to tolerate ﬂuids sufﬁciently to ensure hydration. | Total Oral intolerance or absolute rejection of oral feed, not able to guarantee adequate hydration orally. Required nasogastric and/or intravenous ﬂuids |
|  | Ikibazo mwigogora ry’ibiryo | oya | Kugira apeti nke cyangwa kuruka birikumwe nogukorora | Kuruka burikanya bivanze ninkorora , kwanga ibiryo ariko akanywa bihagije kuburyo ntamwuma afite | Kwanga ibyokurya byose, kuburyo yagira umwuma. Yakeneye sonde yo mugifu cyangwa serumu yo mumutsi |
| 2 | Medical intervention | No | Basic Nasal secretions aspiration, physical examination, trial of nebulized bronchodilators, antipyretics. | Intermediate Oxygen therapy required. Complementary exams were needed (chest X-rays, blood gases, hematimetry.. .). Maintained nebulized therapy with bronchodilators. | High Required respiratory support with positive pressure (either non-invasive in CPAP, BiPAP or high-ﬂow O2; or invasive through endotracheal tube). |
|  | Ubutabazi bwakiganga bwakozwe | Oya | Gusukura amazuru, gusuzuzuma, nebulization n’imiti ifungura ibihaha, imiti igabanya umulilo. | Oxygen iringaniye, ibizami byuzuza nk’amafoto yagatuza, imyuka y’amaraso, nebilization ihoraho y’imiti ifungura agatuza | Gukenera ibyuma bifasha guhumeka nka CPAP, BiPAP, ogisigene nyishi, cg imashini imuhumekera |
| 3 | Respiratory difﬁculty | No | Mild Not in basal situation but does not appear severe.  Wheezing only audible with stethoscope, good air entrance. If modiﬁed Wood Downes, Wang score or any other respiratory distress score is applied, it indicates mild severity. | Moderate Makes some extra respiratory effort (intercostal and/or tracheosternal retraction).  Presented expiratory wheezing audible even without stethoscope, and air entrance may be decreased in localized areas. If modiﬁed Wood Downes, Wang score or any other respiratory distress score is applied, it indicates moderate severity. | Severe Respiratory effort is obvious. Inspiratory and expiratory wheezing and/or clearly decreased air entry. If modiﬁed Wood Downes, Wang score or any other respiratory distress score is applied, it indicates high severity. |
|  | Ibibazo muguhumeka | Oya | Guhumeka nabi byoroheje ariko wheezing zumvikana kuri stethoscope.  Niba indi score yakoresheje nayo yerekanye guhumeka nabi byoroheje. | Guhumeka nabi biringaniye ariko akoresha inyama zo mugatuza.  Wheezing zumivikana na stetoscope mugihe cyo gusohora umwuka. Cyangwa indi score yakoreshejwe yerekanye guhumeka nabi biri murugero. | gukoresha imbaraga nyinshi ngo ahumeke bigaragarira buri wese.  Wheezing mukwinjira no gusohoka ku mwuka  Umwuka muke winjira mubihaha.  Indi score yerekanye guhumeka nabi bikabije. |
| 4 | Respiratory frequency | Normal  < 2 m: 40–50  2–6 m: 35–45  6-12m: 30–40 12-24m:25–35 24-36m: 20–30 | Mild or occasional tachypnea Presented episodes of tachypnea, well tolerated, limited in time by self-resolution or response to secretion aspiration or  nebulization. | Prolonged or recurrent tachypnea Tachypnea persisted or recurred despite secretion aspiration and/or nebulization with bronchodilators. | Severe alteration Severe and sustained tachypnea. Very superﬁcial and quick breath rate. Normal/low breath rate with obvious increased respiratory effort and/or mental status affected. Orientative rates of severe tachypnea:  < 2 m: > 70 bpm  2–6 m: > 60 bpm  6-12m: >55 bpm  12-24m: >50 bpm  24-36m: >40 bpm |
|  | Inshuro zo guhumeka | bizima <2 m : 40-  Hagati ya m 2-6:35-45  Hagati ya m 6-12:30-40  Hagati ya m 12-24:25-35  Hagati ya m 24-36 : 20-30 | Byoroheje cyangwa guhumeka vuba rimwe narimwe. Byoroheje,byo byikijije cyangwa byakize nyuma yo gusukura amazuru cyangwa nebilizasiyo. | Imara igihe kirekire cyangwa guhumeka nabi burikanya cyangwa bihoraho nyuma yo gusukura amazuru cyangwa nebilization n’imiti ifungura ibihaha. | Guhumeka nabi bikabije kandi bihoraho.  Guhumeka atitsa kandi vuba cyane.  Guhumeka bisanzwe cyangwa gake hamwe n’ibimenyetso byerekanako ari gukoresha imbaraga nyinshi ngo ahumeke cyangwa gutakaza ubwenge.  Ibipimo byoguhumeka nabi bikabije  Munsi ya m 2 >70 bpm  Hagati ya m 2-6 >60 bpm  Hagati ya m 6-12 >55 bpm  Hagati ya m 12-14 >50bpm  Hagati ya m 24-36 >40bpm |
| 5 | Apnea | No |  |  | Yes At least one episode of respiratory pause medically documented or strongly suggested through anamnesis. |
|  | Guhagarika guhumeka | Oya |  |  | Nibura inshuro imwe yoguhagarika guhumeka yanditswe. |
| 6 | General Condition | Normal | Mild Not in basal situation, child was mildly uncomfortable but does not appear to be in a severe condition, not impress of severity. Parents are not alarmed. Could wait in the waiting room or even stay at home. | Moderate Patient looks ill, and will need medical exam and eventually further complementary exams and/ or therapy. Parents are concerned. Cannot wait in the waiting room. | Severe Agitated, apathetic, lethargic. No need of medical training to realize severity.  Parents are very concerned. Immediate medical evaluation and/or intervention were required. |
|  | Uko agaragara muri rusange | Ni bizima | Ntabwo ameze neza buhoro ntanubwo agaragara nkurembye.  Ababyeyi ntakibazo bafitye barategerejye bashoraga no kwigumira murugo. | Ameze nkurwaye murugero kandi akeneye gusuzumwa nibindi bizamini cyangwa kuvurwa.  Ababyeyi barahangayitse.  Ntibashobora gutegereza mu cyumba cyogutereza. | Afite amahane, yacitse integer, ari gutakaza ubwenge.  Ntabumenyi bukenewe ngo umenyeko arembye.  Ababyeyi barahangayitse cyane.  Yahawe ubutabazi bwakiganga n’ubuvuzi bwihuse. |
| 7 | Fever | No | Yes, mild Central T < 38.5°C | Yes, moderate Central T > 38.5°C |  |
|  | Umulilo | Oya | Yego, umulilo muke < 38.5°C | Yego , umulilo uringaniye > 38.5°C |  |

(m = months) (m= amezi)

TOTAL SCORE (Umubare wose hamwe) = /21
