# Supplementary material for: Field testing two existing, standardized respiratory severity scores (LIBSS and ReSViNET) in infants presenting with acute respiratory illness to tertiary hospitals in Rwanda – a validation and inter-rater reliability study
Source: PLoS One. 2021 Nov 4;16(11):e0258882. doi: 10.1371/journal.pone.0258882 (PMC8568200; doi:10.1371/journal.pone.0258882)
Supplement: Attachment — Submitted filename: Field testing two existing standardised respiratory severity scores.docx [file pone.0258882.s006.docx]

Field testing two existing standardised respiratory severity scores (LIBSS and

ReSViNET) in infants presenting with acute respiratory illness to tertiary hospitals in Rwanda – a validation and inter-rater reliability study

General comment

The authors have embarked on researching an important topic that is pertinent. In a continent that still carries a huge burden of deaths secondary to respiratory illness, the area covered by this research is imminently relevant. I commend the authors for this effort.

ABSTRACT

I would suggest that the ‘introduction’ contain some sort of rationale for the study

INTRODUCTION

Line 89. Please check if this is indeed LIC or if it is supposed to be LMIC’s. Please note that these mean different things. For example, while South and Malawi are both LMICs, South Africa is Upper-Middle income, while Malawi is Low income…South Africa and India are not LIC

MATERIALS AND METHODS

Line 131. So, to be clear, the children were selected on the basis of having respiratory distress. This would mean that the bases on which they were selected overlapped with the scoring system that will access the severity of their illness, right? This is important later given what the limitations are: the sample taken may not be appropriate to tell us how the tools will fare in detecting severe disease in unselected children (and in primary care levels, children arrive unselected).

Table 1. It may be useful to add columns that indicate the ranges of points for each item in each score.

Line 199. I have struggled to find these odd ratios in the paper. Did I miss them?

Line 200. It is not clear as to what variables the model was adjusted for. What were these confounders? (I will return to this in the results section).

Lines 207-209. I am not entirely sure that doing correlation of two scores that have such a large overlap of components makes any sense at all…. In fact one does not need to collect data to figure out that they will almost be guaranteed to be correlated (as long as apnoea means the same thing in both scores, the fact that scores are allocated slightly differently should have little impact on correlation). In fact the correlation might potentially be seen as almost colinear from basic principles. I can therefore accept correlation of either score to the same but independent outcome, but not to each other… I hope my discomfort with this analysis makes sense (ReSVinet=70% of LIBBS’s components while 50% is the reverse) .

NB. Would it not make sense to compare the severity of each score (a categorical analysis) to clinical severity as indicated in lines 193-194 and see how each ‘agree’ with this??

RESULTS

NB. As the study is about the two scoring methods, I expected to see a summary of the scores generated from these two, then see how they perform against clinical severity as mentioned above

Lines 219-221: 107-8=99, however the data used in the rest of the paper as well as Fig 1 suggest that 7 (and not 8) were excluded.

Line 228 – SD used for the first time here. Suggest writing it in full at first mention. By the way, I would have to confess that it is rather unusual for a number of the continuous variables reported here to be ‘normally’ distributed enough to be acceptably summarised with means and SDs. Were all these data tested for normality (either formally or graphically). Specifically with age, my suggestion would be to summarise this using months rather than days. It is just easier for the reader to immediately comprehend the age of the cohort that way. Hardly do people measure children’s age as days outside the neonatal period.

NB. While, I really appreciate that for this cohort of 100 children, as long as we are referring to proportions of the total, the number will always equal the percentage, it is otherwise good practice to always report both number and percentage [n(%)] in the text. When part of the report includes subgroup proportions, not having numbers to which the percentages refer gets very confusing.

Table 2.

- Table includes ‘social group’ which is undefined
- ‘Vaccination’ what does No and Yes mean?
- Maternal education: What does high and low mean??
- ‘Median length of stay’ but result indicate some sort of standard deviation??

Table 3. By the way, the use of a Kappa statistic is coming here for the first time being absent in methods.

Line 248. I think it should read Table 4 not 3

NB. When it comes to tables and figures, make sure all acronyms are explained in the figure/table legends. E.g. acronyms such as ‘ICC’ in figures..

Table 4. What is the difference between the ‘others’ of “Bronchiolitis and others” and ‘Other’?

Table 5.

- Already alluded to in Methods, I am not sure what is adjusted for here
- Would it be possible to show the data for each binary outcome?
- Secondly, in the case of adjustment being done one would need to show both adjusted and unadjusted estimates
- Length of hospital stay: I can see the others working as ‘binary’ in a Yes-No gold standard to generate ROCs, but how was this variable used as ‘gold’ standard for ROC??
- Some things in the table legend don’t seem to belong to it: Cronbach, SPR

DISCUSSION

My sense is that the section on treatment (Lines 300-314) is an unwarranted digression that important as it is has little to do with the theme of the paper. If there is a need for it, it really needs to be brief

Lines 338-340. I can’t see anything in table 6 indicating the detrimental effect of apnoea to reliability. Maybe if there is anything of the sort the authors could highlight this in the text in the results section before discussing it here.

Lines 343-345; Could the authors not have gone around this issue by doing stratified analysis with just bronchiolitis to see if they find results similar to those in HIC?

CONCLUSION

Are the authors suggesting that the two scores yield similar results? It just seems to me that table 3 suggests that one score misses half of severe cases (or maybe one wrongly doubles the number of severe cases??) This is worrying and needs explanation, but can not be addressed unless the two scores were assessed against a single ‘gold’ standard as suggested previously rather than to each other.
