# Supplementary material for: Field testing two existing, standardized respiratory severity scores (LIBSS and ReSViNET) in infants presenting with acute respiratory illness to tertiary hospitals in Rwanda – a validation and inter-rater reliability study
Source: PLoS One. 2021 Nov 4;16(11):e0258882. doi: 10.1371/journal.pone.0258882 (PMC8568200; doi:10.1371/journal.pone.0258882)
Supplement: Attachment — Submitted filename: Reviewer comments- PLoS one 20-20016.docx [file pone.0258882.s005.docx]

**Reviewer comments: PONE-D-20-20016**

Field testing two existing standardised respiratory severity scores (LIBSS and ReSViNET) in infants presenting with acute respiratory illness to tertiary hospitals in Rwanda, a validation and inter-rater reliability study

**Overall comments**

The paper could benefit from English language editing. It is hard to follow and there are a number of grammatical errors. The main objective of the study is to assess two scores of respiratory distress from HIC one exclusively for bronchiolitis in infants. There is no clear motivation as to why the authors chose not to compare with other tools from LMIC settings taking into account high levels of malnutrition in their population. The patient selection and the motivation for item selection is not clearly articulated. There is no clear impression given to the context of the study. The inclusion and exclusion criteria and limited and the methodology is lacking in defining a number of variables that have been discussed in the document. There is no explanation why some sites failed to recruit any participants. There major limitation is the lack of uniformity of disease classification, 13% of study sample mentioned as other. The representation of the data requires major revisions and the discussion is limited in its academic discussion of the findings and does not seek to address the primary objective and secondary objective.

1. The tables and figures are not comprehensively captioned and there are mistakes when the author makes references to some tables. There are two (Table 1s) in the text.
2. The authors do not use the discussion to elaborate fully on their results they mention that findings are interesting and does try to elaborate, compare and make arguments for or against.
3. References are made to a PhD thesis that is not available in print, textbooks where pages and chapters are not mentioned, references to studies that were apparently done in the UK but when looked up are somewhere else, conclusive remarks supported by comparison to studies with different methodologies (compares apples with oranges).
4. The reporting of numbers is not the same everywhere at times uses 3 decimal points then uses 2, the results in abstract are not the same as those in the tables in results.
5. The introduction is fair but for the discussion, the author has not tried to put create a followable argument, there are a lot of headings with paragraphs that do not have an introduction, body and conclusion, there are just thoughts put under a heading. So the gist of the article is lost.
6. The paper argues for use of a tool developed in resource-rich but does not explain why? e.g. What's wrong with ones developed in LMICs? Also if it will be used to use in different resp illnesses it's no longer a validation study but an adaptation or modified tool. or further development of the tool.   If the study compared its use in bronchiolitis only it would validate the tool.  I am not convinced about the validation argument.

**Methodology**

- Bias: participant selection would be those with severe disease if no self-referral system- how can this be applicable to patients with mild or moderate disease
- How were the participants selected: this is not clear ??
- Other relevant exclusions??
- Table 1; for ease of reading useful to have similar markers for both scores in same line so easy for reader to see which parameters are dissimilar. Also two tables named Table 1.
- There is no explanation of what is meant by Social Group (Ubedehe) High (3 & 4),Low (1 & 2), what levels of schooling etc for parents.
- What vaccination was asked about, what is the EPI program in Rwanda?

Results

- The final number stated is 100 but only 99 were analyzed as there were 107 and 8 exclusions? “from eight infants could not be used due to data collection being incomplete or inadequate”
- Table 1 missing after characteristics no explanation of what data is presented
- Table 1: 28 required HDU and ICU but 13 seem to have received interventions in HDU or PICU
- Lack of consistency with decimal places, confusing results and explanation of key in tables and figures

Discussion

- There is no logical flow to the discussion and this fails to give a detailed academic discussion on the results of the study and the context.

Conclusion: considering the study size, variable disease profiles of children I think the conclusion is slightly over-reaching in the abstract, the conclusion in the main document more modest.
